# One Pathway, Two Cyclic Non-Ribosomal Pentapeptides: Heterologous Expression of BE-18257 Antibiotics and Pentaminomycins from *Streptomyces cacaoi* CA-170360

**DOI:** 10.3390/microorganisms9010135

**Published:** 2021-01-08

**Authors:** Fernando Román-Hurtado, Marina Sánchez-Hidalgo, Jesús Martín, Francisco Javier Ortiz-López, Daniel Carretero-Molina, Fernando Reyes, Olga Genilloud

**Affiliations:** Fundación MEDINA, Avenida del Conocimiento 34, 18016 Granada, Spain; fernando.roman@medinaandalucia.es (F.R.-H.); jesus.martin@medinaandalucia.es (J.M.); javier.ortiz@medinaandalucia.es (F.J.O.-L.); daniel.carretero@medinaandalucia.es (D.C.-M.); fernando.reyes@medinaandalucia.es (F.R.); olga.genilloud@medinaandalucia.es (O.G.)

**Keywords:** pentaminomycin, BE-18257, heterologous expression, Cas9-assisted targeting of chromosome segments (CATCH) cloning, non-ribosomal peptide synthetases (NRPS), secondary metabolites, *Streptomyces*

## Abstract

The strain *Streptomyces cacaoi* CA-170360 produces the cyclic pentapeptides pentaminomycins A–H and BE-18257 A–C, two families of cyclopeptides synthesized by two non-ribosomal peptide synthetases encoded in tandem within the same biosynthetic gene cluster. In this work, we have cloned and confirmed the heterologous expression of this biosynthetic gene cluster, demonstrating that each of the non-ribosomal peptide synthetases present in the cluster is involved in the biosynthesis of each group of cyclopeptides. In addition, we discuss the involvement of a stand-alone enzyme belonging to the Penicillin Binding Protein family in the release and macrocyclization of the peptides.

## 1. Introduction

Cyclic peptides are one of the most important chemical classes of biomolecules with potential therapeutic applications. The cyclic polypeptide chain is formed by amide bonds between proteinogenic or nonproteinogenic amino acids, with a structure that confers reduced conformational flexibility, resistance to exo- and endo-peptidases, increased cell permeability and better biological activities compared with their linear counterparts. Consequently, these molecules show, in general, low toxicity, good binding affinity and target selectivity [1,2].

Bacterial cyclic peptides exhibit a wide variety of biological activities. Best examples of largely used cyclic peptides as therapeutic agents are the antibiotics gramicidin, vancomycin, daptomycin, and polymyxin B [3,4,5]. In recent years, new bacterial bioactive cyclopeptides have been reported, such as pargamicins A–D [6,7], cyclotetrapeptides cyclo-(Leu-Pro-Ile-Pro) and cyclo-(Tyr-Pro-Phe-Gly) [8], the cyclic pentapeptides BE-18257 A–D [9,10] and the pentaminomycins A–E [11,12,13]. Cyclic pentapeptides BE-18257 A–D were first isolated from *Streptomyces* sp. 7338 as endothelin receptor antagonists [9,10]. The pentaminomycins are cyclic pentapeptides that possess the common core sequence Val-Trp-N^δ^-OHArg and show distinct biological activities. Pentaminomycin A has antimelanogenic activity against alpha-melanocyte stimulating hormone (α-MSH)-stimulated B16F10 melanoma cells [11] and pentaminomycin C is active against Gram-positive bacteria but not against Gram-negative bacteria [12]. Both pentaminomycins C and D act as autophagy inducers on HEK293 cells [13].

Recently, Kaweewan et al. [12] and Hwang et al. [13] proposed a biosynthetic gene cluster (BGC) for the production of pentaminomycins C–E and the cyclic pentapeptides BE-18257 A–B. The proposed cluster harbors regulatory, transport-related and biosynthetic genes, including cytochromes P450 and two non-ribosomal peptide synthetases (NRPS), each of them predicted to synthesize one type of pentapeptide. The cluster lacks any gene harboring a thioesterase (TE) domain but contains a stand-alone enzyme belonging to the Penicillin Binding Protein (PBP) family, that, similar to what has been demonstrated for SurE in the biosynthesis of surugamide [14,15,16], is proposed to act as a trans-acting TE cyclizing both BE-18257 A–B and pentaminomycins C–E [12,13].

**Figure 1:**
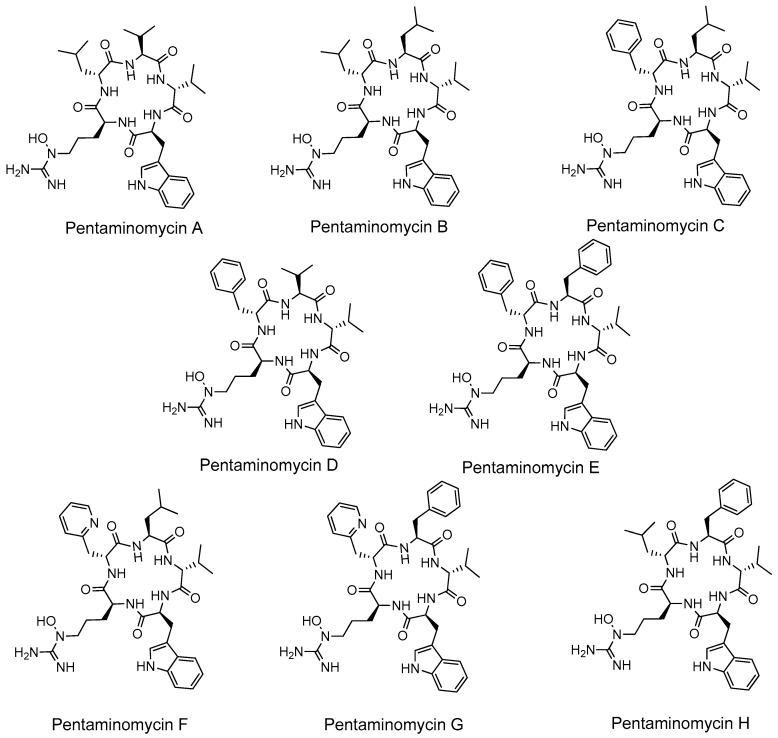
Structures of pentaminomycins A–H.

**Figure 2:**
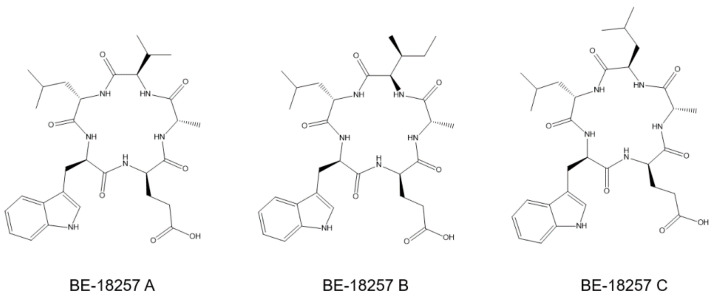
Structures of BE-18257 A–C antibiotics.

The strain *Streptomyces cacaoi* CA-170360 from MEDINA’s microbial collection produces pentaminomycins A–H (Figure 1) and the cyclic pentapeptides BE-18257 A–C (Figure 2) [17]. This strain has also been recently described as the producer of cacaoidin, the founding member of class V lanthipeptides (lanthidins) [18,19]. In this work, we demonstrate that pentaminomycins A–H and BE-18257 A–C are synthesized in this strain by a pathway highly similar to that described recently in other *S. cacaoi* strains [12,13]. To that end, we have cloned and heterologously expressed both the complete BGC and a partial pathway that lacks the NRPS encoding pentaminomycins. In the last case, only BE-18257 antibiotics are detected, confirming the involvement of each NRPS in the biosynthesis of the respective cyclopentapeptides and the putative involvement of the pathway-located PBP-type TE in the cyclization of both types of compounds.

## 2. Materials and Methods

### 2.1. Bacterial Strains and Plasmids

Strain *Streptomyces cacaoi* CA-170360 from Fundación MEDINA’s culture collection was isolated from the rhizosphere of a specimen of *Brownanthus corallinus*, in the region of Namaqualand (South Africa). Electrocompetent NEB 10-β *E. coli* (New England BioLabs, Ipswich, MA, USA), *E. coli* ET12567 (LGC Standards, Manchester, NH, USA) and *E. coli* ET12567/pUB307 (generously provided by J.A. Salas) were employed throughout plasmids transformation and conjugation processes. Vector pCAP01 was used for the cloning of the BGCs. This plasmid is a *S. cerevisiae*/*E. coli*/actinobacteria shuttle, kanamycin-resistant vector with a site-specific φC31 integrase which allows the incorporation of the cloned cluster to the genome of heterologous hosts [20]. *Streptomyces albus* J1074 was used as heterologous host [21] and was kindly provided by J. A. Salas.

### 2.2. Growth and Culture Conditions

Culture media composition is described in the Supplementary Information. *Streptomyces cacaoi* CA-170360 was cultured in ATCC-2 liquid medium and grown on an orbital shaker at 28 °C, 220 rpm and 70% relative humidity. For the OSMAC (one strain many compounds) approach, CA-170360 was cultured in six different liquid media (YEME, R2YE, KH4, MPG, FR23 and DEF-15) and at three different times (7, 14 and 21 days). *E. coli* strains were routinely cultured in LB broth Miller (Sigma, St. Louis, MO, USA) (37 °C, 250 rpm), Difco LB agar Lennox (37 °C, static). Intergeneric conjugations were carried out on solid MA medium and exconjugants were grown on antibiotic-supplemented MA plates. Antibiotics were added when required for selection of transformants at the following final concentrations: kanamycin (50 μg/mL), nalidixic acid (25 μg/mL), chloramphenicol (25 μg/mL). For heterologous expression, MPG and R2YE media were used and the recombinant strains were incubated for 14 days on an orbital shaker at 28 °C, 220 rpm and 70% relative humidity.

### 2.3. Identification of cpp Cluster from Strain CA-170360 Whole Genome Sequence

The genome sequence of *Streptomyces cacaoi* CA-170360 [19] was analyzed by antiSMASH 5.1.2 [22] in order to find the biosynthetic gene cluster responsible for the production of pentaminomycins A–H and BE-18257 A–C. The *cpp* BGC sequence is available in the National Center for Biotechnology Information (NCBI) database under accession GenBank number MW038823.

### 2.4. Cloning and Heterologous Expression of the cpp Gene Cluster

The *cpp* cluster was cloned by CATCH (Cas9-Assisted Targeting of CHromosome), where a Cas9 endonuclease cleaves a large BGC guided by RNA templates [23]. Two types of cloning were performed in this work: one including the NRPS responsible to produce BE-18257 A-C and another one with both NRPS involved in the production of BE-18257 A–C and pentaminomycins A–H.

CRISPy-web tool (http://crispy.secondarymetabolites.org/) was employed to design 20 nt target sequences close to a PAM (Protospacer-Adjacent Motif) sequence “NGG” [24] that is the target where Cas9 endonuclease cuts. Based on these sequences, the necessary primers are listed in Table S1.

An overlapping PCR was carried out using three oligos, one target-specific oligo (Penta1-sgRNA, Penta2-sgRNA or Penta3-sgRNA) containing the target sequence and a T7 promoter and two universal oligos (sgRNA-F and sgRNA-R) in order to get the three Penta-sgRNAs needed for this study. Q5 High-Fidelity polymerase from New England BioLabs (Ipswich, MA, USA) was employed for this PCR. HiScribe T7 Quick Yield RNA synthesis kit (New England Biolabs, Ipswich, MA, USA) was used for the in vitro transcription and the products were purified by phenol/chloroform extraction and isopropanol precipitation, as Jian and Zhu described in their protocol [25].

*Streptomyces cacaoi* CA-170360 was cultured in ATCC-2 at 28 °C, 220 rpm and 70% relative humidity to later be embedded in low-melting agarose plugs where the in-gel Cas9 digestion was performed. The genomic DNA of the strain was extracted within the plugs using lysozyme, proteinase K and washing buffers, and once the genome was isolated, in-gel digestion with Cas9 nuclease from *S. pyogenes* (New England BioLabs, Ipswich, MA, USA) was performed taking two plugs of agarose, a cleavage buffer (100 mM HEPES pH 7.5, 750 mM KCl, 0.5 mM EDTA pH 8, 50 mM MgCl_2_, DEPC-treated water) and the sgRNAs, and incubating at 37 °C for 2 h. After the digestion, the agarose plugs were melted with a GELase treatment and the already digested DNA was recovered with an ethanol precipitation. The pCAP01 vector was previously amplified with the oligos pCAP01-Penta1-F/pCAP01-Penta1-R and pCAP01-Penta2-F/pCAP01-Penta2-R (Table S1) to get 30 nt overlapping ends. Then, the Cas9-cleavaged BGCs were cloned in the corresponding amplified vector by Gibson Assembly using a 2× Gibson Assembly Master Mix (New England BioLabs, Ipswich, MA, USA) and incubating at 50 °C for 1 h. The Gibson products, pCPP1 and pCPP2, were transformed into electrocompetent NEB 10-β *E. coli* cells. Plasmids pCPP1 and pCPP2 from isolated colonies were validated by restriction digestion with HindIII and NdeI.

As pCPP1 and pCPP2 contain the kanamycin-resistant marker, two triparental intergeneric conjugations were made using *E. coli* NEB 10-β /pCPP1 or *E. coli* NEB 10-β/pCPP2 and non-methylating Cm^R^ Km^R^ *E. coli* ET12567/pUB307 as donor strains, and spores of *S. albus* J1074 as recipient strain. For the negative control, *E. coli* NEB 10-β/pCAP01 and *E. coli* ET12567/pUB307 were used as donor strains.

Five positive transconjugants from each conjugation, together with the negative control and the wild-type strain *S. cacaoi* CA-170360, were grown on liquid MPG and R2YE media for 14 days at 28 °C, and then acetone extracts from the cultures were obtained.

### 2.5. Extraction and Detection of BE-18257 Antibiotics and Pentaminomycins

Cultures of the recombinant strains *S. albus* J1074/pCPP1 and *S. albus* J1074/pCPP2, together with the negative control harboring empty pCAP01 vector and the original *S. cacaoi* CA-170360 as positive control, were subjected to extraction by liquid–liquid partition with acetone 1:1, stirring at 220 rpm for 2 h. Once dried under a nitrogen atmosphere, the residue was resuspended in 20% DMSO/water and the resulting microbial extracts were analyzed by LC-HRESI-TOF.

## 3. Results and Discussion

### 3.1. Production of Cyclic Pentapeptides by Strain CA-170360

**Figure 3:**
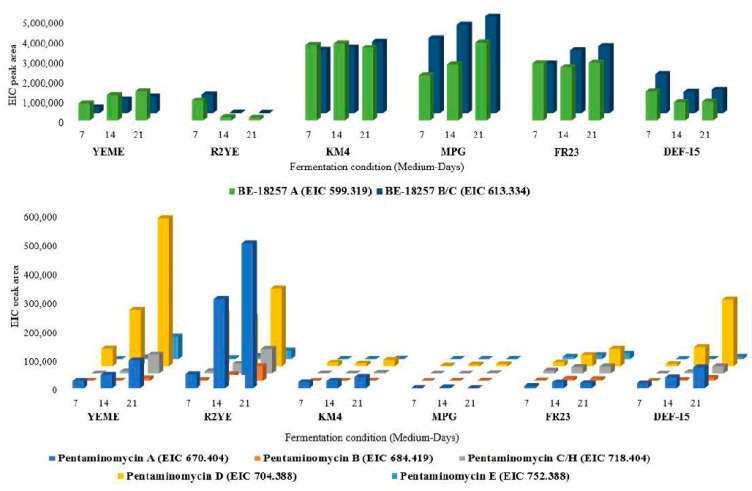
Production of BE-18257 antibiotics (top) and pentaminomycins A–E and H (bottom) by strain *S. cacaoi* CA-170360 in six different media at three different times (7, 14 and 21 days). The average extracted ion chromatogram (EIC) peak area from triplicate culture extracts of the strain CA-170360 is represented.

In our continuous effort to search for novel compounds, the strain *Streptomyces cacaoi* CA-170360 was shown to produce the cyclic pentapeptides BE-18257 A–C and, to a much lesser extent, the recently described pentaminomycins A–H after liquid fermentation in MPG medium for 13 days [17]. We followed an OSMAC approach [26] to identify the best production conditions of both families of cyclopeptides. The analysis included a total of six production media (YEME, R2YE, KM4, MPG, FR23 and DEF-15) and three fermentation times (7, 14 and 21 days). Production of BE-18257 A–C was the highest in KM4, MPG and FR23 media whereas the detection levels of pentaminomycins were very low. Pentaminomycins A–E were mostly produced in YEME and R2YE and required long incubations of 14 and 21 days, although the production was still very low. Pentaminomycin H [17] coelutes with pentaminomycin C in the LC-HRMS analytical conditions employed. Therefore, the peak might contain either isomers or a mixture of both. The production of the new pentaminomycins F and G is described in reference [17]. Interestingly, in these conditions, BE-18257 A–C were produced in very small amounts, suggesting that these media ensure the biosynthesis of pentaminomycins to the detriment of the BE-18257 molecules (Figure 3). To our knowledge, CA-170360 is the first strain reported to produce all pentaminomycins described to date and the three BE-18257 compounds. The strain *Streptomyces* sp. RK88-1441 was shown to produce pentaminomycins A and B but no BE-18257 antibiotics [11] while only pentaminomycin C and BE-18257 A were isolated from the strain *Streptomyces cacaoi* subsp. *cacaoi* NBRC 12748^T^ [12]. Finally, Hwang et al. [13] reported the production of pentaminomycins C–E and BE-18257 A–B from *Streptomyces* sp. GG23. These strains may have the capacity to synthesize all the pentaminomycins and BE-18257 antibiotic variants detected in strain CA-170360, but most probably the culture conditions used did not ensure the production of all the analogs.

### 3.2. Identification of the cpp Gene Cluster from the Whole Genome Sequence of Strain CA-170360

**Figure 4:**
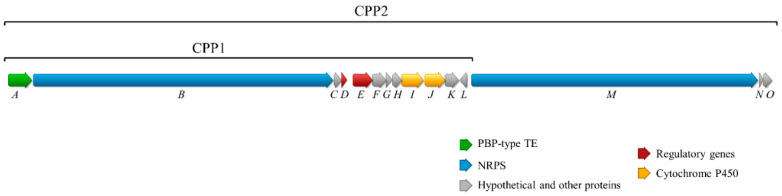
*cpp* biosynthetic gene cluster. It contains two non-ribosomal peptide synthetases (NRPS) genes (blue), one Penicillin Binding Protein (PBP)-type thioesterase (TE) gene (green), two cytochromes P450 (yellow), two regulatory genes (red) and other genes with unknown functions (grey). The two fragments cloned by CATCH into vector pCAP01 are indicated: the 28.7 Kb CPP1 fragment contains the PBP-type TE gene (*cppA*), the NRPS1 gene (*cppB*) and the genes present between NRPS1 and NRPS2 (*cppC-cppL*); the 48 Kb CPP2 fragment includes the above described 28.7 Kb fragment and the NRPS2 (*cppM*), *cppN* and *cppO* genes.

The whole genome sequence of *Streptomyces cacaoi* CA-170360 [19] was analyzed with antiSMASH [22] and 31 putative BGCs, including NRPS, polyketide synthase (PKS) and ribosomally synthesized and post-translationally modified peptides (RiPPs), among others, were predicted. One of the BGCs from contig 1 (*cpp* cluster) was identified as the putative pathway for the synthesis of both BE-18257 A-C and pentaminomycins A–H (GenBank number MW038823). The *cpp* gene cluster contains 15 open reading frames (ORFs) coding for proteins with the proposed functions shown in Table 1 and Figure 4.

**Figure 5:**
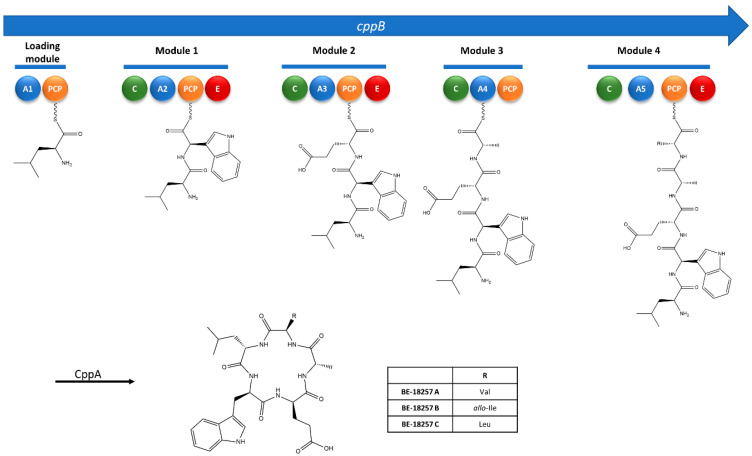
Proposed biosynthetic pathway for the BE-18257 A–C antibiotics with the non-ribosomal peptide synthetase CppB modular organization. A1-A5, adenylation domains; PCP, peptidyl carrier protein; C, condensation domain; E, epimerase domain; CppA, PBP-type TE.

The gene organization and amino acid incorporation is highly similar to those previously proposed for these BGCs in strains NBRC 12748^T^ and GG23 [12,13], with two NRPS genes, each containing five adenylation (A) domains. The first NRPS gene (*cppB*) contains three epimerization (E) domains and a sequence of amino acids corresponding to Leu (A1), Trp (A2), Leu/Ser (A3), Ala (A4) and Val/Leu (A5). The three E domains are located in the second, third and fifth modules, and they would be involved in the isomerization of an L- to D- amino acid, resulting in the final sequence L-Leu, D-Trp, D-Leu/Ser, L-Ala, D-Val/Leu, which is in accordance with the amino acid sequence of BE-18257 A-C (L-Leu, D-Trp, D-Glu, L-Ala, D-Val/D-*allo*-Ile/D-Leu) (Figure 5).

**Figure 6:**
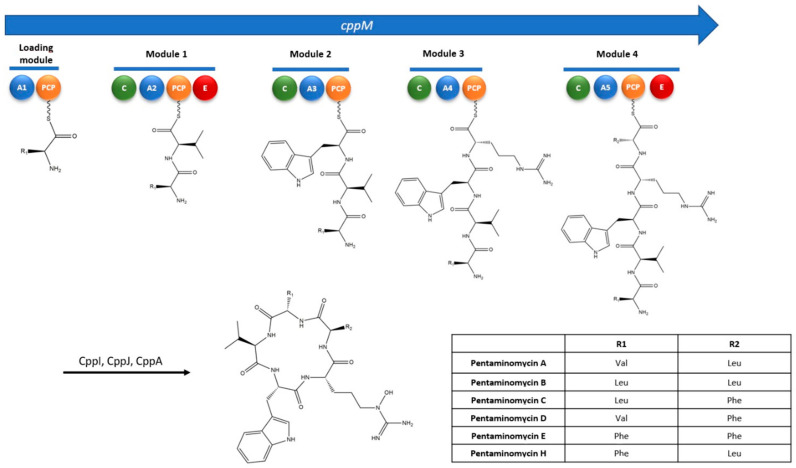
Proposed biosynthetic pathway for the pentaminomycins A–H with the non-ribosomal peptide synthetase CppM modular organization. A1-A5, adenylation domains; PCP, peptidyl carrier protein; C, condensation domain; E, epimerase domain; CppI and CppJ, cytochromes P450; CppA, PBP-type TE.

Therefore, these results suggest that the first NRPS gene (*cppB)* may be involved in the biosynthesis of BE-18257 A–C antibiotics. Then, cyclization would complete the biosynthesis of the molecules. On the other hand, the second NRPS gene (*cppM*) contains two E domains and the sequence of amino acids incorporated would be Val/Leu/Phe (A1), Val (A2), Trp (A3), Arg (A4) and Leu/Phe (A5). The two E domains are located in the second and fifth modules, so the final amino acid sequence would be L-Val/Leu/Phe, D-Val, L-Trp, L-Arg, D-Leu/Phe, which agrees with the amino acid sequence of pentaminomycins A–E and H (L-Val/L-Leu/L-Phe, D-Val, L-Trp, L-N5-OH-Arg, D-Leu/D-Phe) (Figure 6). Subsequent modifications such as hydroxylation and cyclization would complete the biosynthesis of the pentaminomycins. However, the *cpp* cluster also lacks a TE domain to release and cyclize the pentapeptides but contains a PBP-type TE stand-alone protein (*cpp*A) that may be involved in the release and cyclization of the peptide chains of both BE-18257 antibiotics and pentaminomycins, as it was proposed by Kaweewan et al. [12] and Hwang et al. [13]. In fact, it has been recently described that SurE, a stand-alone enzyme belonging to the PBP family, is involved in the release and macrocyclization of two different surugamides (B and F) encoded in a single gene cluster [14,15,16,27]. This PBP-type TE has been also reported in other NRPS pathways such as those of desotamide [28], ulleungmycin [29], noursamycin [30], curacomycin [31] or mannopeptimycin [32].

The *cpp* cluster includes two ORFs (*cppI* and *cppJ*) encoding cytochrome P450 enzymes, which have been suggested to be involved in the N-hydroxylation of arginine to form 5-OH-Arg in pentaminomycins, as previously suggested [12,13]. The pathway also contains regulatory genes and other genes of unknown function (Table 1, Figure 4).

### 3.3. Cloning and Heterologous Expression of the cpp Gene Cluster

To demonstrate that the identified *cpp* cluster is involved in the biosynthesis of both BE-18257 A-C and pentaminomycins A–H, we separately cloned two different fragments of the BGC by Cas9-assisted targeting of chromosome segments (CATCH) cloning [23], a main approach to clone long microbial genomic sequences, into vector pCAP01 [33]. This method uses in-gel RNA-guided Cas9 nuclease digestion of bacterial DNA, which is subsequently ligated with cloning vector by Gibson assembly [25]. The first genome sequence cloned was a 28.7 Kb fragment containing the PBP-type TE gene (*cppA*), the NRPS1 gene (*cppB*) and the genes present between NRPS1 and NRPS2 (*cppC-L*) to obtain pCPP1; the second one was a 48 Kb fragment including all the genes supposed to be required for the biosynthesis of both antibiotics; this was the previously described 28.7 Kb fragment and the NRPS2 gene (*cppM*), together with two genes encoding hypothetical proteins downstream of NRPS2 (*cppN-O*), to obtain pCCP2 (Figure 4).

The plasmids pCPP1 and pCPP2 were transformed into *E. coli* NEB 10-β competent cells. Clones were checked by restriction analysis, and one of the clones harboring pCPP1 and another one harboring pCPP2 were selected to perform intergeneric conjugations. Since pCPP1 and pCPP2 contain the kanamycin-resistant marker, we could not directly transform non-methylating Cm^R^ Km^R^ *E. coli* ET12567/pUB307. Thus, we performed two triparental intergeneric conjugations using *E. coli* NEB 10-β/pCPP1 and ET12567/pUB307 or *E. coli* NEB 10-β/pCPP2 and ET12567/pUB307 as donor strains, and spores of *S. albus* J1074 as recipient strain. For the negative control, a triparental conjugation was also made using *E. coli* NEB 10-β/pCAP01 and ET12567/pUB307 as donor strains and the same recipient strain. Transconjugants were checked by PCR with primers BLAC check-F and BLAC check-R (Table S1) to confirm the integration of the cloned BGCs into the chromosome of *S. albus* J1074.

Five positive transconjugants from each conjugation, together with the negative control and the wild-type strain *S. cacaoi* CA-170360, were grown in liquid MPG and R2YE media (to favor the detection of BE-18257 antibiotics and pentaminomycins, respectively) for 14 days at 28 °C, and acetone extracts from the cultures whole broths were prepared. After removing the solvent, the residue was resuspended in 20% DMSO/water and analyzed by LC-HRESI-TOF.

**Figure 7:**
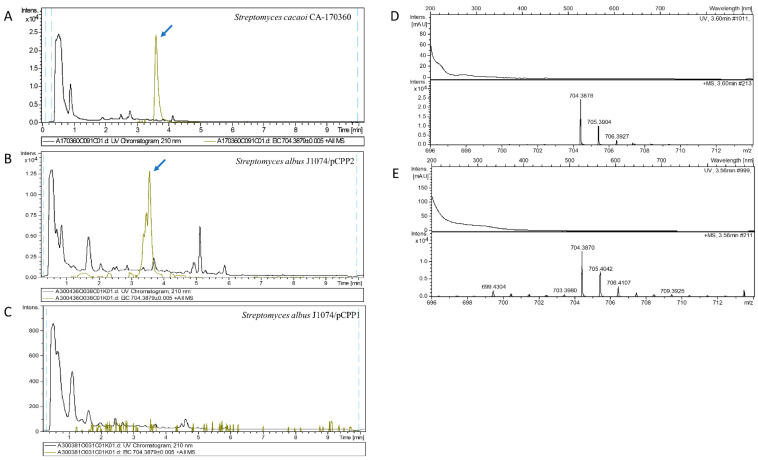
Pentaminomycin D production. Left. Chromatograms of UV absorbance at 210 nm and extracted ion m/z = 704.3879 ± 0.005, C_36_H_50_N_9_O_6_^+^ of pentaminomycin D (blue arrow) from original producing strain *Streptomyces cacaoi* CA-170360 (**A**) and the heterologous producing strains *Streptomyces albus* J1074/pCPP2 (**B**) and *Streptomyces albus* J1074/pCPP1 (**C**). Right. Experimental UV and positive mass spectra from C_36_H_50_N_9_O_6_^+^ (calculated value: 704.3879) adduct from original producing strain *Streptomyces cacaoi* CA-170360 (**D**) and the heterologous producing strain *Streptomyces albus* J1074/pCPP2 (**E**). No UV or mass spectra were obtained with the heterologous producing strain *Streptomyces albus* J1074/pCPP1 as it did not carry the NRPS gene required for the production of pentaminomycins. The chromatograms showing the production of the rest of pentaminomycins are depicted in Figures S3–S5.

The analysis of extracts from pCPP1 and pCPP2 transconjugants confirmed the presence of peaks at 3.46 min and 3.77 min, coincident with the retention time of elution of the three BE-18257 A–C isolated from the CA-170360 strain (Figures S1 and S2). The detection levels of the BE-18257 A–C molecules in the pCPP1 transconjugants (which lacked the pentaminomycins NRPS gene) were much higher than in the pCPP2 transconjugants (which also carried the pentaminomycins NRPS gene). The analysis of the pCPP2 transconjugants also confirmed the presence of peaks coincident with the retention time of elution of the pentaminomycins C/H, D and E, isolated from CA-170360, which were absent in the pCPP1 transconjugants (Figure 7, Figures S3 and S4).

The correlation between the UV spectrum, exact mass and isotopic distribution between the BE-18257 and pentaminomycins from *S. cacaoi* CA-170360 and the components isolated from the transconjugants *S. albus*/pCPP1 and *S. albus*/pCPP2 (Figure 7 and Figures S1–S4) unequivocally confirmed that they corresponded to BE-18257 A–C in the case of *S. albus*/pCPP1 and to BE-18257 A–C and pentaminomycins C/H, D and E in the case of *S. albus*/pCPP2. In the pCPP2 transconjugants, we detected ions suggesting the presence of pentaminomycins A and B but given the low production levels of these compounds, we could not obtain proper mass spectra (Figure S5). The detection levels of all the cyclopentapeptides in the heterologous hosts was lower than in the *S. cacaoi* strain, in which the pentaminomycins were already poorly produced. Consequently, the productions of pentaminomycins in the heterologous host *S. albus*/pCPP2 were still at the limit of detection from most of the compounds. These results clearly demonstrate that the first NRPS gene (*cppB*) is responsible for the biosynthesis of BE-18257 antibiotics, and that the second NRPS gene (*cppM*) synthesizes pentaminomycins. The results also suggest that the cluster-located PBP-type TE is involved in the cyclization of both compounds, and that the *cpp* BGC can be considered an atypical case in which two types of independent compounds are processed by the same enzyme.

The genome of *S. cacaoi* CA-170360 also contains some genes related to tryptophan biosynthesis downstream from the second NRPS gene (*cppM*), as it has been already described by Hwang et al. [13] for strain GG23. As both pentaminomycins and BE-18257 contain tryptophan in their structures, it has been proposed that they may share the Trp biosynthetic genes to incorporate this amino acid [13]. However, our results clearly show that those genes are not required to incorporate Trp in the cyclic pentapeptides, since they were not included in the fragment cloned into pCPP1 and in pCPP2, and the pentaminomycins and BE-18257 were still produced. This indicates that the tryptophan, as well as the rest of the amino acids, are obtained from the primary metabolism amino acid pool.

### 3.4. Genome Mining of cpp-like BGCs

**Figure 8:**
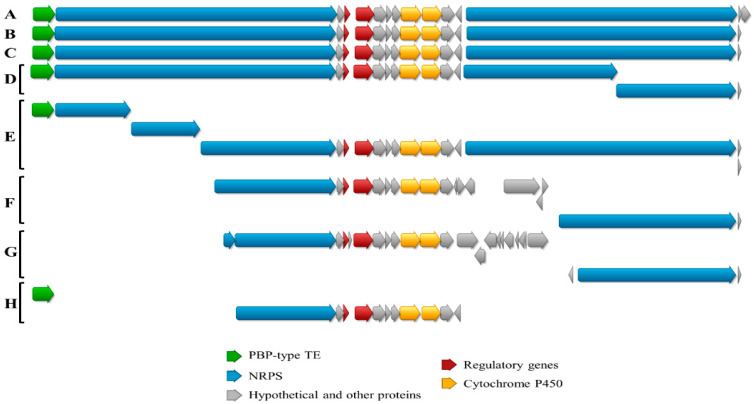
Schematic representation of the alignment of *cpp* BGC from *Streptomyces cacaoi* CA-170360 and the homologous genome sequences found in NCBI. A, *S. cacaoi* CA-170360; B, *S. cacaoi* NHF165; C, *S. cacaoi* DSM 40057; D, *S. cacaoi* NRRL B-1220; E, *S. cacaoi* OABC16; F, *Streptomyces* sp. NRRL S-1868; G, *Streptomyces* sp. NRRL F-5053 and H, *S. cacaoi* NBRC 12748. Due to the high fragmentation in some of these *S. cacaoi* genomes, the corresponding BGC was found in different contigs and was not complete.

A tblastn search of the NRPS1 and NRPS2 protein sequences against both nucleotide and Whole Genome Sequence (WGS) databases from NCBI showed that the *cpp* cluster is also present in some genomes described in Figure 8. Moreover, the pathway is only present in strains belonging to *Streptomyces cacaoi* species: *S. cacaoi* NHF165, *S. cacaoi* DSM 40057, *S. cacaoi* subs. *cacaoi* NRRL-1220, *S. cacaoi* OABC16, *Streptomyces* sp. NRRL S-1868, *Streptomyces* sp. NRRL F-5053 and *S. cacaoi* NBRC 12748 (Figure 8).

The *pen* cluster described by Hwang et al. [13] in the strain *Streptomyces* sp. GG23, which has been also identified as a strain of *Streptomyces cacaoi,* has not been included in this analysis because the sequence is not yet available. Nevertheless, the comparison of the homologies described in the *pen* and in the *cpp* clusters clearly shows that they are highly similar. This indicates, as was described for the cacaoidin cluster [19], that the *cpp* cluster is highly conserved within members of this species and is another excellent example of the biosynthesis of a specialized metabolite that could be used as a species-specific trait [34].

## 4. Conclusions

We have shown that BE-18257 antibiotics and pentaminomycins can be produced heterologously from a single BGC (*cpp*) containing two independent NRPS genes, *cppB* and *cppM*, encoding, respectively, each type of pentapeptide, and one PBP-type TE stand-alone protein (CppA) that is proposed to be involved in the release and cyclization of both families of compounds. We have also demonstrated that the downstream genes related to tryptophan biosynthesis, an amino acid that is present in all the cyclic pentapeptides, are not necessary for their production. Furthermore, our bioinformatic analysis suggests that the *cpp* cluster might be a species-specific trait since it was only found in the genomes of all publicly available *Streptomyces cacaoi* strains and not in other species. Despite the lack of similar BGCs found in genome sequence databases beyond *Streptomyces cacaoi* species, this work opens the door to identify additional tandem biosynthetic genes organized within a single BGC to ensure the biosynthesis of related families of compounds, as well as the biosynthesis of new analogs of both BE-18257 antibiotics and pentaminomycins.

## Figures and Tables

**Table 1:** Closest BLAST homolog for each ORF in *cpp* biosynthetic gene cluster.

ORF	Length (aa)	Closest BLAST Match (Organism) GenBank Reference	Identity (%)/Similarity (%)
*cppA*	502	Hypothetical protein DEH18_05445 (*Streptomyces* sp. NHF165) QHF93414.1	99/99
*cppB*	6187	Non-ribosomal peptide synthase/polyketide synthase (*Streptomyces cacaoi*) WP_158102276.1	99/99
*cppC*	172	MULTISPECIES: DUF2975 domain-containing protein (*Streptomyces*) WP_030891799.1	100/100
*cppD*	102	Helix-turn-helix domain-containing protein (*Streptomyces cacaoi*) WP_149564434.1	99/99
*cppE*	420	MULTISPECIES: sensor histidine kinase (*Streptomyces*) WP_051857187.1	99/100
*cppF*	280	Hypothetical protein SCA03_67000 (*Streptomyces cacaoi* subsp. *cacaoi*) GEB54149.1	100/100
*cppG*	131	MULTISPECIES: DUF742 domain-containing protein (*Streptomyces*) WP_030891786.1	100/100
*cppH*	186	MULTISPECIES: ATP/GTP-binding protein (*Streptomyces*) WP_030891784.1	100/100
*cppI*	451	MULTISPECIES: cytochrome P450 (*Streptomyces*) WP_030891781.1	100/100
*cppJ*	431	Cytochrome P450 (*Streptomyces cacaoi*) WP_086815207.1	99/99
*cppK*	271	3-hydroxybutyryl-CoA dehydratase (*Streptomyces cacaoi* subsp. *cacaoi*) GEB54144.1	99/99
*cppL*	141	MULTISPECIES: hypothetical protein (*Streptomyces*) WP_141275837.1	100/100
*cppM*	5901	Non-ribosomal peptide synthase/polyketide synthase (*Streptomyces* sp. NHF165) WP_159784853.1	99/99
*cppN*	69	MULTISPECIES: hypothetical protein (*Streptomyces*) WP_030890086.1	100/100
*cppO*	175	MULTISPECIES: hypothetical protein (unclassified *Streptomyces*) WP_030890089.1	99/100

## Data Availability

The data presented in this study are openly available in NCBI, GenBank reference number [MW038823]. Publicly available datasets were analyzed in this study. These data can be found in NCBI, GenBank reference numbers [CP029241.1, JABELW000000000.1, MUBL00000000.1, VSKT00000000.1, JOGD00000000.1, JOHT00000000.1 and BJMM00000000.1].

## Supplementary Materials

The following are available online at https://www.mdpi.com/2076-2607/9/1/135/s1: Culture media composition, Figure S1: BE-18257 A production chromatogram, Figure S2: BE-18257 B/C production chromatogram, Figure S3: Pentaminomycin C/H production chromatogram, Figure S4: Pentaminomycin E production chromatogram, Figure S5: Pentaminomycins A and B production chromatograms, Table S1: Oligonucleotides used in this work.
