# Supplementary material for: One Pathway, Two Cyclic Non-Ribosomal Pentapeptides: Heterologous Expression of BE-18257 Antibiotics and Pentaminomycins from Streptomyces cacaoi CA-170360
Source: Microorganisms. 2021 Jan 8;9(1):135. doi: 10.3390/microorganisms9010135 (PMC7827011; doi:10.3390/microorganisms9010135)
Supplement: Supplementary file 1 [file microorganisms-09-00135-s001.pdf]

microorganisms

Supporting On-line Information

ATCC-2

- Soluble starch 20 g/L
- Glucose 10 g/L
- NZ Amine Type E 5 g/L
- Meat extract 3 g/L
- Peptone 5 g/L
- Yeast extract 5 g/L
- Calcium carbonate 1 g/L

pH adjusted to 7

YEME

- Yeast extract 3 g/L
- Bacto-peptone 5 g/L
- Oxoid malt extract 3 g/L
- Glucose 10 g/L
- Sucrose 340 g
- $MgCl_2 \cdot 6H_2O$ 2mL/L

R2YE

- Yeast extract 5 g/L
- Sucrose 103 g/L
- K_2SO_4 0.25 g/L
- $MgCl_2 \cdot 6H_2O$ 10.12 g/L
- Glucose 10 g/L
- Casamino acids 0.1 g/L
- KH_2PO_4 0.5 % 1 mL
- $CaCl_2 \cdot 2H_2O$ 3.68 % 8 mL
- L-proline 20 % 1.5 mL
- TES buffer 5.73 % adjusted to pH7.2 10 mL
- Trace element solution 0.2 mL
- NaOH 1N 0.5mL

Trace element solution:

- $ZnCl_2$ 40 mg/L
- $FeCl_3 \cdot 6H_2O$ 200 mg/L
- $CuCl_2 \cdot 2H_2O$ 10 mg/L
- $MnCl_2 \cdot 4H_2O$ 10 mg/L
- $Na_2B_4O_7 \cdot 10H_2O$ 10 mg/L
- $(NH_4)_6Mo_7O_{24} \cdot 4H_2O$ 10 mg/L

KM4

- Glucose 4 g/L
- Yeast extract 4 g/L
- Malt extract 10 g/L
- $CaCO_3$ 2 g/L

MPG

- Glucose 10 g/L
- Millet 20 g/L

- Cottonseed flour 20 g/L
- MOPS 20 g/L

pH adjusted to 7

FR23

- Glucose 5 g/L
- Soluble starch from potato 30 g/L
- Cottonseed flour 20 g/L
- Cane molasses 20 g/L

pH adjusted to 7

DEF-15

- Sucrose 40 g/L
- $ClNH_4$ 2 g/L, Na_2SO_4 2 g/L
- K_2HPO_4 1 g/L
- $Cl_2Mg{\cdot}6H_2O$ 1 g/L
- Trace elements 1 mL
- $CaCO_3$ 2 g/L

pH adjusted to 7
Trace elements:

- $MnCl_2{\cdot}4H_2O$ 0.1 g/100 mL
- $ZnCl_2$ 0.1 g/100 mL
- $FeCl_2{\cdot}4H_2O$ 0.1 g/100 mL
- NaI 0.05 g/100 mL

MA

- MOPS 21 g/L
- Glucose 5 g/L
- Yeast extract 0.5 g/L
- Beef extract 0.5 g/L
- Casamino acids 1 g/L
- Agar 25 g/L

pH adjusted to 7

Figures

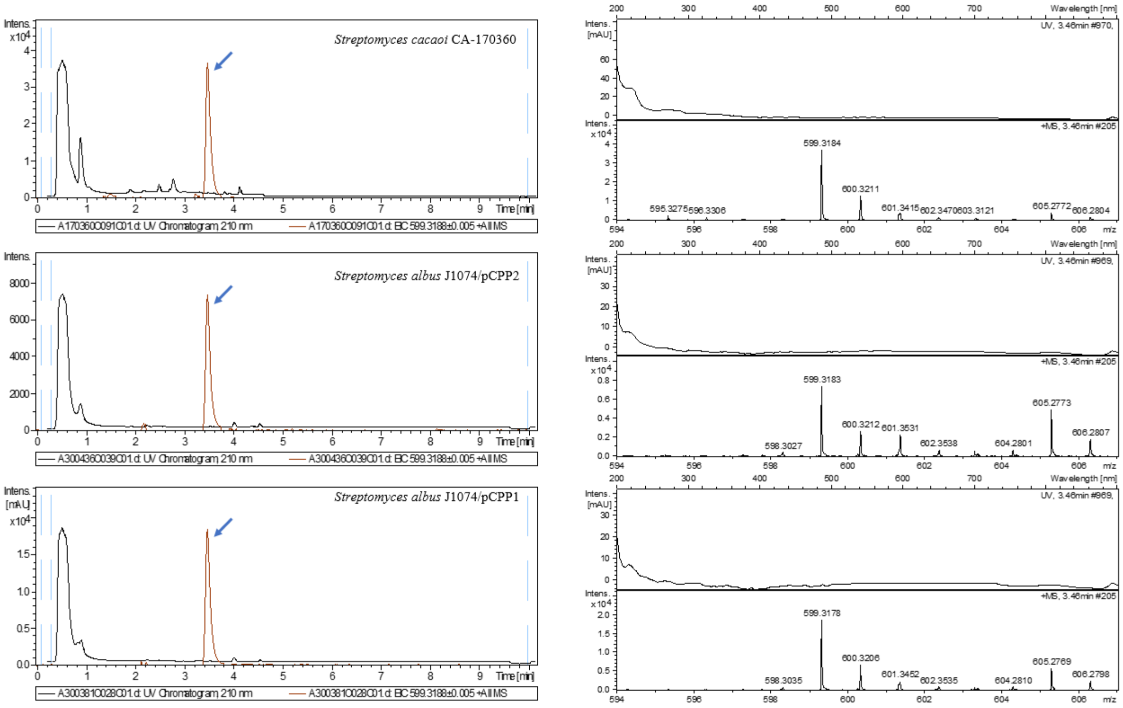

Figure S1. BE-18257 A production. Left. Chromatograms of UV absorbance at 210 nm and extracted ion m/z = 599.3188 ± 0.005, $C_{30}H_{43}N_6O_7^+$ of BE-18257 A (blue arrows) from original producing strain *Streptomyces cacaoi* CA-170360 (top) and the heterologous producing strains *Streptomyces albus* J1074/pCPP2 (middle) and *Streptomyces albus* J1074/pCPP1 (bottom). Right. Experimental UV and positive mass spectra from $C_{30}H_{43}N_6O_7^+$ (calculated value: 599.3188) adduct from original producing strain *Streptomyces cacaoi* CA-170360 (top) and the heterologous producing strains *Streptomyces albus* J1074/pCPP2 (middle) and *Streptomyces albus* J1074/pCPP1 (bottom).

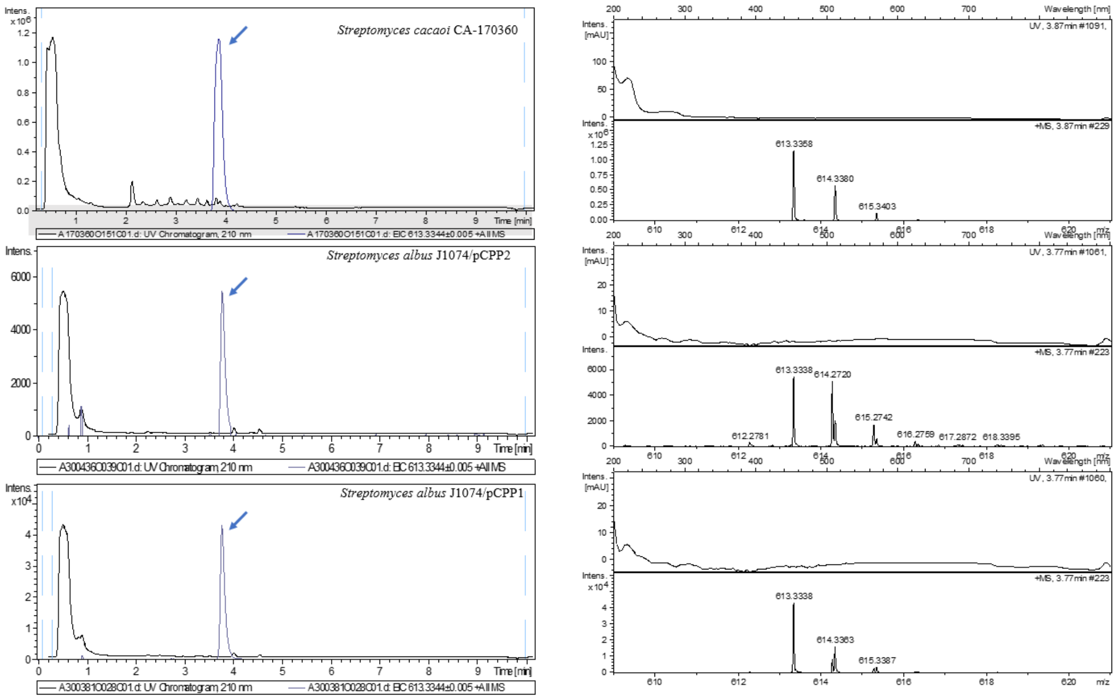

Figure S2. BE-18257 B/C production. Left. Chromatograms of UV absorbance at 210 nm and extracted ion m/z = 613.3344 ± 0.005, $C_{31}H_{45}N_6O_7^+$ of BE-18257 B/C (blue arrows) from original producing strain *Streptomyces cacaoi* CA-170360 (top) and the heterologous producing strains *Streptomyces albus* J1074/pCPP2 (middle) and *Streptomyces albus* J1074/pCPP1 (bottom). Right. Experimental UV and positive mass spectra from $C_{31}H_{45}N_6O_7^+$ (calculated value: 613.3344) adduct from original producing strain *Streptomyces cacaoi* CA-170360 (top) and the heterologous producing strains *Streptomyces albus* J1074/pCPP2 (middle) and *Streptomyces albus* J1074/pCPP1 (bottom).

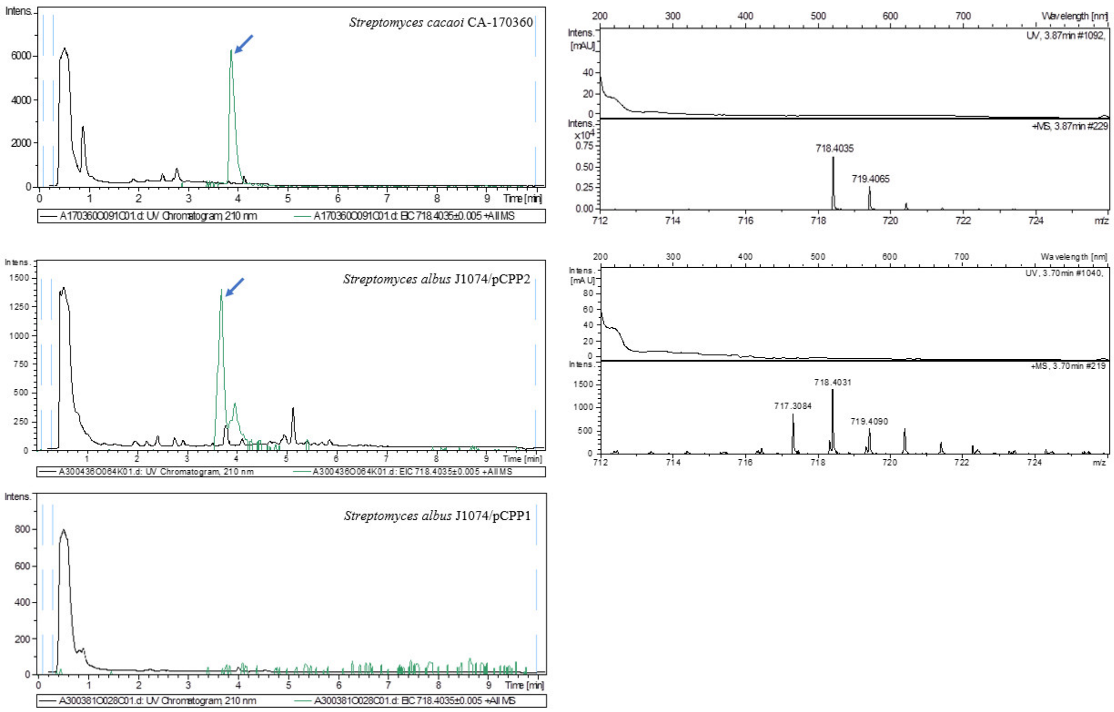

Figure S3. Pentaminomycin C/H production. Left. Chromatograms of UV absorbance at 210 nm and extracted ion m/z = 684.4192 ± 0.005, $C_{34}H_{54}N_9O_6^+$ of pentaminomycin C/H (blue arrows) from original producing strain *Streptomyces cacaoi* CA-170360 (top) and the heterologous producing strains *Streptomyces albus* J1074/pCPP2 (middle) and *Streptomyces albus* J1074/pCPP1 (bottom). Right. Experimental UV and positive mass spectra from $C_{34}H_{54}N_9O_6^+$ (calculated value: 684.4192) adduct from original producing strain *Streptomyces cacaoi* CA-170360 (top) and the heterologous producing strain *Streptomyces albus* J1074/pCPP2 (middle). No UV or mass spectra was obtained with the heterologous producing strain *Streptomyces albus* J1074/pCPP1 as it did not carry the NRPS gene required for the production of pentaminomycins.

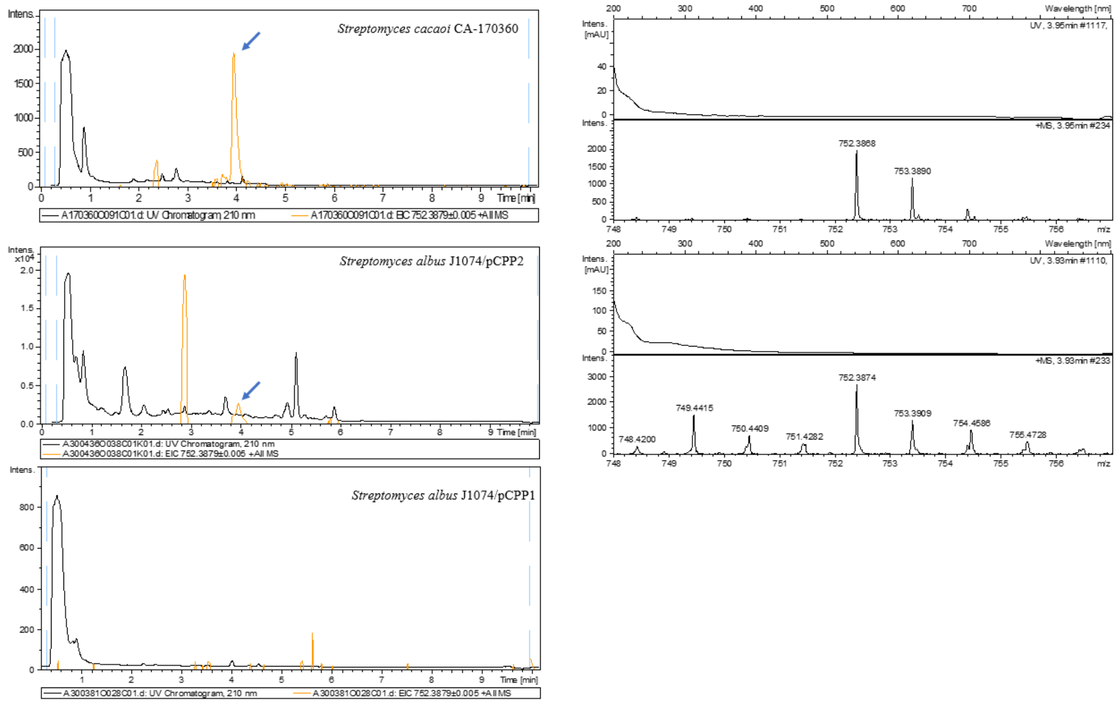

Figure S4. Pentaminomycin E production. Left. Chromatograms of UV absorbance at 210 nm and extracted ion m/z = 752.3879 ± 0.005, $C_{40}H_{50}N_9O_6^+$ of pentaminomycin E (blue arrows) from original producing strain *Streptomyces cacaoi* CA-170360 (top) and the heterologous producing strains *Streptomyces albus* J1074/pCPP2 (middle) and *Streptomyces albus* J1074/pCPP1 (bottom). Right. Experimental UV and positive mass spectra from $C_{40}H_{50}N_9O_6^+$ (calculated value: 752.3879) adduct from original producing strain *Streptomyces cacaoi* CA-170360 (top) and the heterologous producing strain *Streptomyces albus* J1074/pCPP2 (middle). No UV or mass spectra was obtained with the heterologous producing strain *Streptomyces albus* J1074/pCPP1 as it did not carry the NRPS gene required for the production of pentaminomycins.

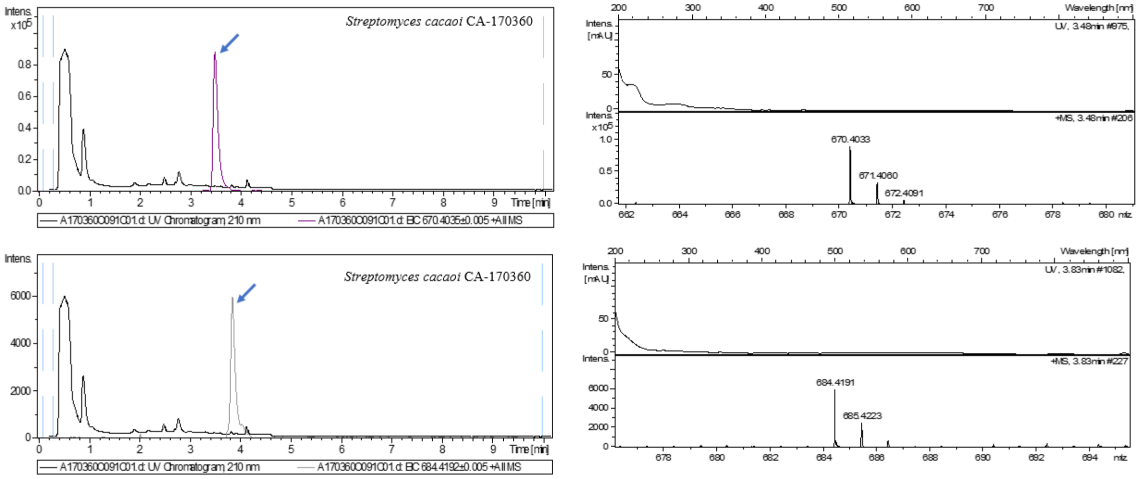

Figure S5. Pentaminomycins A and B production. Left. Chromatogram of UV absorbance at 210 nm and extracted ion m/z = 670.4035 ± 0.005, $C_{33}H_{52}N_9O_6^+$ of pentaminomycin A (top, blue arrow) and extracted ion m/z = 684.4192 ± 0.005, $C_{34}H_{54}N_9O_6^+$ of pentaminomycin B (bottom, blue arrow) from original producing strain *Streptomyces cacaoi* CA-170360. Right. Experimental UV and positive mass spectra from $C_{33}H_{52}N_9O_6^+$ (calculated value: 670.4035) adduct (top) and $C_{34}H_{54}N_9O_6^+$ (calculated value: 684.4192) adduct (bottom) from original producing strain *Streptomyces cacaoi* CA-170360. Proper mass or ultraviolet spectra of pentaminomycins A and B could not be obtained in the heterologous hosts. .

Tables

Table S1. Oligonucleotides used in this work.

Oligonucleotide	Sequence (5′-3′)
**Penta1-sgRNA**	TAATACGACTCACTATAGATGATCCAGAATCCGTGCTTGTTTTAGAGCTAGAAATAGCAA
**Penta2-sgRNA**	TAATACGACTCACTATAGACCCCAGACTTCAGCGTTTGGTTTTAGAGCTAGAAATAGCAA
**Penta3-sgRNA**	TAATACGACTCACTATAGGAACTGAAGGCACAACCAAAGTTTTAGAGCTAGAAATAGCAA
**sgRNA-F**	GTTTTAGAGCTAGAAATAGCAAGTTAAAATAAGGCTAGTC
**sgRNA-R**	AAAAGCACCGACTCGGTGCCACTTTTTCAAGTTGATAACGGACTAGCCTTATTTTAACT
**pCAP01-Penta1-F**	AGGCTAGTCAGGGGTACCGGGCCCCTCAAATCGAGACTTGAGGTACCTGT
**pCAP01-Penta1-R**	TCGGAAAGCGGCTGAAGGTCTCTCCCAAGCTCGAGGTTACTAGTCGATCT
**pCAP01-Penta2-F**	AAGGCTAGTCAGGGGTACCGGGCCCCTCAATCGAGACTTGAGGTACCTGT
**pCAP01-Penta2-R**	GGCCAACTGGCCTGCTACCTGCGCCATTTGTCGAGGTTACTAGTCGATCT
**BLAC check-F**	CCAACTCCTCGAACAGCT
**BLAC check-R**	CTGCTCAGCCACACCG
